# Patterns and associated factors of electrocardiographic abnormality among type 2 diabetic patients in Amhara National Regional State Referral Hospitals, Ethiopia: a multicenter institution-based cross-sectional study

**DOI:** 10.1186/s12872-022-02661-2

**Published:** 2022-05-19

**Authors:** Deresse Sinamaw, Mihret Getnet, Mohamed Abdulkadir, Kassa Abebaw, Mohammed Ebrahim, Mengistie Diress, Yonas Akalu, Adugnaw Ambelu, Baye Dagnew

**Affiliations:** 1 Department of Biomedical Science, School of Medicine, College of Medicine and Health Sciences, Debre Markos University, P. O. Box 269, Debre Markos, Ethiopia; 2 Department of Human Physiology, School of Medicine, College of Medicine and Health Sciences, University of Gondar, P. O. Box 196, Gondar, Ethiopia; 3 Department of Internal Medicine, School of Medicine, College of Medicine and Health Sciences, University of Gondar, P. O. Box 196, Gondar, Ethiopia; 4 Department of Biomedical Science, School of Medicine, College of Medicine and Health Sciences, Meda Welabu University, P. O. Box 247, Meda Welabu, Ethiopia

**Keywords:** Electrocardiographic abnormalities, Types 2 diabetes mellitus, Ethiopia

## Abstract

**Background:**

Cardiovascular diseases are the most causes of mortality and morbidity among diabetes mellitus (DM) patients. Electrocardiographic (ECG) changes are common in the early course of the disease. Little is known about the electrocardiographic abnormalities among type 2 DM patients in Ethiopia. This study determined the overall prevalence, its patterns, and the associated factors of ECG abnormalities among people living with T2DM in Amhara National Regional State referral hospitals, Ethiopia.

**Methods:**

A multicenter institution-based cross-sectional study was conducted from 01 April to 30 May 2021. A simple random sampling and systematic sampling techniques were employed to select the referral hospitals and study participants, respectively. A digital electrocardiograph was used to measure the ECG parameters and the other data were collected using an interviewer-administered questionnaire. Epi-data version-4.6 and Stata-14 were used for data entry and statistical analysis, respectively. The descriptive statistics were presented with tables and graphs. A binary logistic regression model was fitted to identify associated factors of ECG abnormality. In the final model, statistical significance was decided at *p*≤0.05, and the strength of association was indicated using an adjusted odds ratio with 95% CI.

**Results:**

Two-hundred and fifty-eight participants (response rate = 99.6%) were included for the analysis. The prevalence of overall ECG abnormality was 45% (95% CI: 39, 51%). On the basis of the electrocardiographic patterns, 57 (21.1%; 95% CI: 14.6, 32.6%) were presented with T-wave abnormality, 36 (14%; 95% CI: 10.1, 18.8%) left axis deviation, and 24 (9.3% [6.3, 13.5%]) sinus tachycardia. Higher monthly income (> 90$) (AOR = 0.51 [0.31, 0.83]), over 10 years duration of DM (AOR = 4.5[1.05, 18.94]), hypertension (AOR = 3.9 [1.6, 9.40]), fasting blood sugar of ≥ 130 mg/dl (AOR = 5.01[2.13, 12.20]), and overweight (AOR = 2.65[1.17, 5.98]) were statistically significant factors of overall ECG abnormality.

**Conclusions:**

Nearly, half of the participants had at least one ECG abnormality. Higher-income, prolonged disease duration, hypertension, higher fasting blood sugar, and overweight were significantly associated with ECG abnormality. The findings of this study suggest the need to institute routine ECG screening for all T2DM patients to reduce ECG abnormalities and further complications.

## Background

Cardiovascular diseases (CVD) are the most prevalent causes of mortality and morbidity among people living with DM through micro-vascular and macro-vascular complications [1–4]. In people with DM, CVD is responsible for 24–30% of hospitalization and about one-third of deaths [5] from which coronary artery disease (CAD) accounts for 75–90% of deaths [6]. Undiagnosed and long-lasting T2DM patients are at higher risk of microvascular diseases (Diabetic retinopathy, nephropathy, and neuropathy) and macro-vascular diseases i.e. coronary heart disease, peripheral vascular disease, cerebrovascular disease, and above 70% of T2DM patients die due to CVDs [3, 7, 8]. This cardiovascular complication is elevated in T2DM patients as a result of oxidative stress due to hyperglycemia, dyslipidemia, obesity, hypertension, and physical inactivity [8–12].

Electrocardiographic changes appear early in the course of DM including sinus tachycardia, QTc prolongation, QT dispersion, heart rate variability, ST–T changes, and left ventricular hypertrophy [13, 14]. Globally, about 30% of asymptomatic T2DM patients showed ECG abnormalities [14]. In a study on African-American T2DM patients, there was a 60% of ECG abnormality with QTc prolongation (25.5%), T wave changes (22%), LVH (18.5%), sinus tachycardia (15.5%), ischemic heart disease (9%), conduction defect (7%) and ST-T abnormalities (24.3%) [15]. ECG abnormalities among T2DM were 22% in Canada [16], and also in India, 52% of DM patients had ECG abnormalities with axis deviation (6%), chamber enlargement (6%), heart rate abnormalities (8%), bundle branch blocks (8%), and prolonged QT interval (8%) [17]. Though there is limited evidence of ECG abnormalities in Africa, a study in Senegal revealed a 20% prevalence of ECG abnormalities [18].

ECG abnormality is associated with older age, male sex, smoking cigarette, higher BMI, higher fasting glucose, hypertension, and longer duration of DM [15, 18, 19]. A study in the USA showed that ECG abnormality in T2DM is associated with age, DM duration, and sex [15]. In the Netherlands, smoking cigarettes, obesity, fasting glucose greater than 126 mg/dl, and hypertension were significantly associated with ECG abnormalities [19]. In Senegal, higher ECG abnormalities were observed in females [18]. In Nigeria, age and duration of DM were associated with ECG abnormalities [20]. Moreover, a recently published article in Ethiopia (at Jimma Medical Center) showed DM duration above 10 years, being overweight, and not attending formal education were significantly associated with ECG abnormalities [21].

Common measures that have been done to reduce cardiovascular complications were resting ECG examination every 3–5 years for the duration of diabetes > 15 years, > 40 years old, end-organ damage, and more than one cardiovascular disease risk factor [22].

In Ethiopia, at Jimma Medical Center in Oromia Region, the prevalence of ECG abnormality was 61% among apparently healthy adult T2DM patients [21]. Nonetheless, this study was conducted only on healthy adult type 2 diabetic patients. Moreover, it didn’t address common features of ECG abnormalities in T2DM, and there was no similar study conducted in Amhara Region. Therefore, this study aimed to assess the patterns of ECG abnormality and its associated factors among people living with T2DM in selected referral hospitals in Amhara Region.

## Materials and methods

### Study setting, design, and period

This study was conducted from April 01 to May 30, 2021, in selected referral hospitals in the Amhara region i.e. Bahir Dar Felege Hiwot referral Hospital, Debre Tabor referral Hospital, and Debre Markos Referral Hospital. In Amhara Region, seven referral hospitals provide comprehensive diabetic follow-up care. From these three hospitals were selected to conduct this study. First, Debre Markos referral hospital has provided comprehensive diabetic care services to an estimated catchment population size of more than 3.5 million. The diabetic follow-up clinic in Debre Markos Referral Hospital provides service from Monday to Friday for a total of more than 3840 type 2 DM patients currently attending follow-up at this Hospital. Second, Debre Tabor referral hospital is situated in South Gondar, which is 667 km away from Addis Ababa, and 102 km away from Bahir Dar. It provides diabetic care follow-up every day from Monday to Friday for a total of 1634 T2DM patients. Lastly, Felege Hiwot Referral Hospital (FHRH) is found in Bahir Dar City, situated 565 km away from Addis Ababa, and provides comprehensive diabetic care services to an estimated catchment population size of 6 million. It, also, offers diabetic care follow-up every day from Monday to Friday for a total of 3312 T2DM patients.

### Population and eligibility criteria

The source population was all T2DM patients, who had follow-up at the referral hospitals in Amhara National Regional State. The study population was all T2DM patients who came for a follow-up during the study period in the selected referral hospitals in Amhara National Regional State. All T2DM patients who were diagnosed with T2DM, 18 years old and over, and who came for follow-up during the study period were included in the study. However, T2DM patients with a known thyroid disorder and symptoms that suggest thyroid disorder, who were taking beta-blocker drugs, pregnant, and severely ill at the time of data collection were excluded.

### Sample size determination and sampling technique

The required sample size was determined using the single population proportion formula by taking an estimated prevalence of 20% (*p* = 0.2) of ECG abnormality from the study conducted in Senegal [18] with the assumption of 95% CI and 5% margin of error (d = 0.05). Then, by considering oversampling of 5% for unpredictable events, the final sample size was 259. To determine the sample size, we used proportions from a study conducted in Senegal. Because the study conducted in Jimma was published after this study data was collected. In this study, a simple random sampling technique was applied to select three out of seven referral hospitals in Amhara Region. Then, proportional allocation of samples was employed for each selected hospital based on the number of people living with T2DM. The study population in the selected referral hospitals was 1502. Finally, a systematic sampling technique was used to recruit each study participant from the selected referral hospitals after the Kth interval was determined for individual study participants (1502) to each selected hospital by using the formula: K = N/n: (640/110, 550/94, and 312/55 = 6). Then, the starting point was 2, selected from 1–6 randomly by the lottery method. The next participant was selected every 6th interval from follow-up.

### Operational/conceptual definitions

*The electrocardiographic alteration* was identified deviation of ECG pattern from normal sinus ECG pattern based on Minnesota ECG coding criteria [23].

*T wave abnormality* was identified when T-wave is inverted, peaked, or flattened [23].

*Prolonged PR interval* was identified when PR interval was greater than 0.2 s [23].

*A short PR interval means a* PR interval less than 0.12 s [23].

*Prolonged QT Interval was recognized when the time* taken from the start of the Q wave to the end of the T wave; was normal (0.37–0.44 s), prolonged if takes > 0.0.4 s [23].

*Short QT interval when* QT interval was < 0.37 s [23].

*ST-segment depression* is defined as 1mm below the baseline in I, aVL, V6 [24].

*ST-segment Elevation* is defined as 1mm above the baseline in I, aVL, V6 [24].

*Myocardial Infarction* is defined as ST-elevation > 1 mm, which is associated with multi-lead T wave inversion, while ST depression is the opposite lead [37].

*Ischemic heart disease* is defined as multi-lead ST-segment depression > 1 mm, and T wave inversion [20].

*Left atrial enlargement means P* wave duration > 0.12 s in lead I and II with negative portion > 1 mm in depth and 0.04 s in duration [25]. Right atrial enlargement is defined as a tall > 2.5 mm P wave in limb leads [25].

*Left ventricular hypertrophy*: Sokolow Lyon technique (sum of the amplitudes of S wave in V1 and R wave in V5 or V6 ≥ 35 mm) whether male or female and R wave > 11 mm in lead aVL [25].

*Right ventricular hypertrophy* is a right axis deviation of + 110^0^ and more or dominant R wave in V1 > 7 mm tall [25].

*Left axis deviation is from* −30° to −90° in leads I, II, III [26].

*The right axis deviation is from* 120° to − 90° in leads I, II, and III [27].

*Right bundle branch block* is defined as **a** wide QRS complex, secondary R’ wave in lead V1 or V2, or wide S-wave in lead I, V5, and V6 [27].

*Left bundle branch block* is wide QRS complex and deep S-wave in lead v1 and V2 [27].

*Left anterior fascicular block*: Abnormal left axis deviation in the absence of an inferior myocardial infarction or other causes of left axis deviation [27].

*Left posterior fascicular block* was defined as abnormal right axis deviation in the absence of an inferior myocardial infarction or other causes of right axis deviation [27].

*The bi-fascicular block* was defined as the right bundle branch block and the left anterior or posterior fascicular block [27].

*Regular physical exercise* is defined as an adult doing moderate physical exercise 2–5 h per week [28].

*Higher-income* is defined as a person earning above 90 US dollars per month according to Ethiopian income distribution [29].

### Data collection instruments and procedures

The data were collected using an interviewer-administered structured questionnaire by using the World Health Organization (WHO) stepwise standard questionnaire comprising socio-demographic characteristics, anthropometric measurement, and clinical factors by one professional nurse and one supervisor (Nurse) after providing three days of training. We also used a digital electrocardiographic machine (CONTEC ECG1200G) for ECG parameters, blood pressure apparatus for blood pressure, weighing balance for weight, and height scales for height measurements. Electrocardiographic abnormalities-related questionnaires were adopted from previously published articles on ECG abnormalities among T2DM [15, 18, 19]. The ECG measurement was taken by a one trained person in ECG recording, and interpretation of its abnormality was done by a cardiologist.

## Measurement variables

### Electrocardiographic measurement

The study used standard resting 12 lead digital electrocardiographic machines with the model (CONTEC ECG 1200G, made in China) with a paper speed of 25 mm per second calibrated on 1 mv = 10 mm, 0.2 s = 5 mm, where each large box and the small box represent 0.2 and 0.04 s respectively. Measurement was performed at a supine position with a 45-degree inclination after the patient had been asked to expose the chest region and remove electromagnetic objects on his/her body. And, orientation was given for the patients to stop speaking, move, and reduce breathing during ECG recording. Then, ten electrodes (4 limb electrodes at the right arm, left arm, and legs, and, 6 chest electrodes, V1-V6) were placed on the participant’s arms, legs, and chest after the transparent gel was applied, yielding a total of 12 leads [23]. Finally, the Minnesota coding system was used to identify ECG tracings as having an abnormality [27].

### Anthropometric measurement

The weight (kg) was taken with only light clothing to the nearest 0.1 kg by using body scale. Height (m) was taken to the nearest 0.1 cm with subjects standing erect without shoes or headgear by using a metric stadiometer. The body mass index (BMI) was calculated from the formula; BMI = wt/(ht)^2^ (W = Weight in kg, H = Height in meters) [30].

### Blood pressure measurement

A standard sphygmomanometer blood pressure device was used to measure blood pressure in a relaxed sitting position on the left arm parallel to the heart by wearing an appropriately sized cuff without crossing the legs after 5 min of rest was given. Two consecutive BP measurements at a minimum interval of 5 min were obtained, and the average of the two measurements was taken.

### Data quality control

To assure data quality, the questionnaire was pretested at Finote Selam General Hospital, Northwest Ethiopia. Three days of training were given to data collectors about the objective of the study, methods of data collection, and how to obtain information from those selected individuals. Moreover, during the data collection period, the data was reviewed and checked for completeness by the principal investigator and supervisor.

### Study variables

#### Dependent variable

Electrocardiographic abnormality (binary; dichotomized as Yes or No).

#### Independent variables

Age, sex, marital status, residence, educational status, occupation, income, history of heart disease, history of kidney disease, body mass index, fasting blood sugar, blood pressure, duration of diabetes, physical activity, cigarette smoking, khat chewing, type of drug for DM.

### Data processing and analysis

The collected data was checked for completeness and entered into Epi-data version 4.6 and then exported into Stata-14 for analysis. Descriptive statistics like mean, standard deviation (for continuous variables), and frequency with percentage were executed (for categorical variables) based on the nature of the data after checking normal distribution. Association between independent variables and ECG abnormalities was determined using binary logistic regression. Hosmer Lemeshow test was done to test model fitness (*p* > 0.05). Firstly, a bivariable analysis was performed on each of the selected indicators for the dataset. Then, any variable of *p* < 0.2 was entered into a multivariable regression analysis to identify variables that had a statistically significant association with ECG abnormalities (*p* ≤ 0.05). The strength of the association was determined by computing crude odds ratio (COR) and adjusted odds ratio (AOR) with a 95% CI.

## Results

### Socio-demographic and clinical characteristics of the participants

In this study, a total of 258 (males = 133) T2DM patients participated, making a 99.6% response rate. The mean age of the respondents was 56.7( ± 12.7, range = 28–80) years. The mean monthly income was $112.9 (±  $65.6) (Table 1), and the mean duration of DM was 6.6 (± 5.24) years, ranging from 6 months to 25 years (Table 2). The mean fasting blood sugar was 154.4 ± 50.6 mg/dl. One hundred and nine (42.3%) of the respondents were hypertensive, and the mean body mass index (BM) was 25.8 ± 4 kg/m^2^. Hypertension, BMI of 25 kg/m^2^ and above, and DM duration of above 10 years were associated with electrocardiographic abnormality in chi-square analysis. Moreover, there was a mean difference in age, FBS, duration of diabetes, and BMI between T2DM patients with ECG abnormality and without ECG abnormality.

**Table 1:** Socio-demographic characteristics of T2DM patients grouped by ECG abnormalities using chi-square and student t-test among selected Referral Hospitals in Amhara National Regional State, Ethiopia, 2021 (n = 258)

Variables	Categories	Total/mean (SD) (%)	ECG abnormality		X^2^/T-test p-value
			Yes (%)	No (%)	
Sex	Male	133 (51.60)	57 (22.1)	76 (29.4)	*p* = 0.48
	Female	125 (48.40)	59 (22.9)	66 (25.6)	
Age (years)	< 45	69 (26.80)	5 (1.90)	64 (24.80)	*p* < 0.001
	46–54	46 (17.80)	21 (8.20)	25 (9.70)	
	55–64	66 (25.60)	35 (13.60)	31 (12.00)	
	> 65	77 (29.80)	55 (21.30)	22 (8.50)	
Residence	Urban	195 (75.58)	74 (28.7)	121 (47)	*p* < 0.001
	Rural	63 (24.42)	42 (16.3)	21 (8)	
Current marital status	Married	188 (72.87)	74 (28.7)	114 (44.2)	*p* ≤ 0.05
	Unmarried	70 (27.13)	42 (16.3)	28 (10.8)	
Educational status	No formal education	94 (36.43)	58 (22.5)	36 (14)	*p* < 0.001
	1^0^ and 2ndry education	15 (5.81)	8 (3.1)	7 (2.7)	
	College and above	149 (57.76)	50 (19.4)	99 (38.3)	
Occupation	Employed	122 (47.29)	43 (16.7)	79 (30.6)	*p* < 0.01
	Unemployed	136 (52.71)	73 (28.3)	63 (24.4)	
Income (USD)	< $57.34	97 (32.60)	69 (26.7)	28 (10.9)	*p* < 0.01
	$57.34–$103.2	32 (12.40)	17 (6.6)	15 (5.8)	
	> $103.2	129 (50.00)	30 (11.6)	99 (38.4)	
Regular exercise	Yes	42 (16.28)	7 (2.7)	35 (13.6)	*p* < 0.001
	No	216 (83.72)	109 (42.2)	107 (41.5)	

*USD* United states Dollar, *SD* Standard deviation

**Table 2:** Clinical profile of T2DM patients grouped by ECG abnormalities using chi-square and student t-test, among selected Referral Hospitals in Amhara National Regional State, Ethiopia, 2021 (n = 258)

Variables	Categories	Total/mean (SD)	ECG abnormality		X^2^/T-test (*p*-value)
			Yes (%)	No (%)	
Duration of DM (years)	< 2	73(28.2)	11(4.3)	62(24)	*p* < 0.01
	2–5	64(24.8)	20(7.8)	44(17)	
	6–10	64(24.8)	39(15)	25(9.7)	
	> 10	57(22.0)	46(17.)	11(4.3)	
Type of DM drug	No drug	12(4.6)	7(2.7)	5(1.9)	*p* < 0.001
	Oral only	207(80.2)	99(38.4)	108(.41.9)	
	Insulin only	28(10.9)	6(2.3)	22(8.6)	
	Oral and insulin	11(4.3)	6(2.3)	5(1.9)	
History of kidney disease	Yes	13(5.04)	11(4.3)	2(0.7)	*p* < 0.01
	No	245(94.96)	105(40.7)	140(54.3)	
FBS	< 130 mg/dl|	98(37.98)	14(5.4)	84(32.6)	*p* < 0.001
	≥ 130 mg/dl|	160(62.02)	102(39.5)	58(22.5)	
Hypertension	Yes	109(42.25)	84(32.6)	25(9.7)	*p* < 0.001
	No	149(57.75)	32(12.4)	117(45.3)	
BMI (kg/m^2^)	< 25	133(51.55)	32(12.4)	101(39.1)	*p* < 0.001
	25 and above	125(48.45)	84(32.6)	41(15.9)	

*BMI* body mass index, *FBS* fasting blood sugar, *DM* diabetes mellitus, *SD* standard deviation

### Overall ECG abnormality and its patterns among T2DM

The prevalence of overall ECG abnormality among people living with T2DM was 45% (95% CI: 39, 51%). In this study, the most commonly encountered ECG abnormalities were T-wave abnormalities 57 (21.1%), left axis deviation 36 (14%), sinus tachycardia 24 (9.3%), left anterior fascicular block 22 (8.5%), ST-segment depression 17 (6.6%), poor R-wave progression 17 (6.6%), first-degree heart block 16 (6.2%), sinus bradycardia 13 (5%), prolonged QRS wave 12 (4.7%), possible myocardial ischemia 10 (3.9%), left bundle branch block 9 (3.5%), prolonged QT interval 8 (3.1%), possible myocardial infarction 8 (3.1%), LVH 7 (2.7%), and right bundle branch block 5 (1.9%). The median PR interval of the study participants was 0.17 + 0.024 s (Table 3).

**Table 3:** Patterns of ECG abnormalities among T2DM in selected Referral Hospitals in Amhara National Regional State, Ethiopia, 2021 (n = 258)

Variables	Category	Frequency/mean	Percent (%) (95% CI)
ECG abnormality	Abnormal	116	44.96 (39, 51)
	Normal	142	55.04 (48.9, 61)
Heart rate pattern	Normal	221	85.66 (80.8, 89.5)
	Bradycardia	13	5.04 (2.9, 8.5)
	Tachycardia	24	9.30 (6.3, 13.5)
Arrhythmia	Present	38	14.73 (10.9, 19.6)
	Absent	220	85.27 (80.4, 89.1)
Types of arrhythmia	Atrial flutter	9	33.3 (17.5, 54.0)
	Atrial fibrillation	14	51.9 (32.5, 70.1)
	PVCs	4	14.8 (5.3, 35)
P wave duration	Normal	255	98.84 (96.4, 99.6)
	Short	1	0.39 (0.05, 2.7)
	Prolonged	2	0.78 (0.1, 3.0)
T wave abnormality	Normal	199	77.13 (72.8, 82.9)
	Inverted	38	14.73 (10.9, 19.6)
	Flattened	15	5.81 (3.2, 9.0)
	Peaked	4	1.55 (0.5, 4.0)
QRS wave duration	Normal	242	93.80 (91.0, 96.8)
	Short	4	1.55 (0.4, 3.6)
	Prolonged	12	4.65 (2.4, 7.6)
R wave progression	Normal	241	93.4 (91.1, 97.2)
	Poor	17	6.6 (3.2, 8.4)
PR interval	Normal	242	93.80 (90, 96)
	Prolonged	16	6.20 (3.8, 9.9)
QT interval	Normal	246	95.35 (92, 97)
	Short	4	1.55 (0.5, 4.0)
	Prolonged	8	3.10 (1.6, 6.10)
ST-segment	Normal	240	93.02 (89, 95.6)
	Depressed	17	6.59 (4.1, 10.4)
	Elevated	1	0.39 (0.05, 2.7)
Left atrial hypertrophy	Yes	2	0.78 (0.2, 3.1)
	No	256	99.22 (97.99.8)
RAH	Yes	3	1.2 (0.3, 3.6)
	No	255	98.8 (96.4, 99.6)
LVH	Yes	7	2.71 (1.3, 5.6)
	No	251	97.29 (94.4, 98.7)
RVH	Yes	1	0.39 (0.05, 2.7)
	No	257	99.61 (97.0, 99.9)
Possible myocardial ischemia	Yes	10	3.88 (2.1, 7.0)
	No	248	96.12 (93, 98)
Possible MI	Yes	8	3.10 (1.6, 6.1)
	No	250	96.90 (93.9, 98.4)
Axis deviation	Normal	220	85.27 (80, 89)
	Left axis deviation	36	13.95 (10.1, 18.8)
	Right axis deviation	2	0.78 (0.2, 31)
Bundle branch block	No	244	94.57 (91, 96.8)
	RBBB	5	1.94 (0.8, 4.6)
	LBBB	9	3.49 (1.8, 6.6)
Fascicular block	No	232	89.92 (85.6, 93)
	LAFB	22	8.53 (5.7, 12.6)
	LPFB	1	0.39 (0.05, 2.7)
	BIFB	3	1.16 (0,4, 3.6)

*LVH* left ventricular hypertrophy, *RAH* right atrial hypertrophy, *RVH* right ventricular hypertrophy, *RBBB* right bundle branch block, *LBBB* left bundle branch block, *SD* standard deviation, *PVCs* premature ventricular contractions, *LAFB* left anterior fascicular block, *LPFB* left posterior fascicular block, *BIFB* bi-fascicular bock, *MI* myocardial infarction

### Comparison of T wave abnormality and left axis deviation in T2DM with and without hypertension

**Fig. 1:**
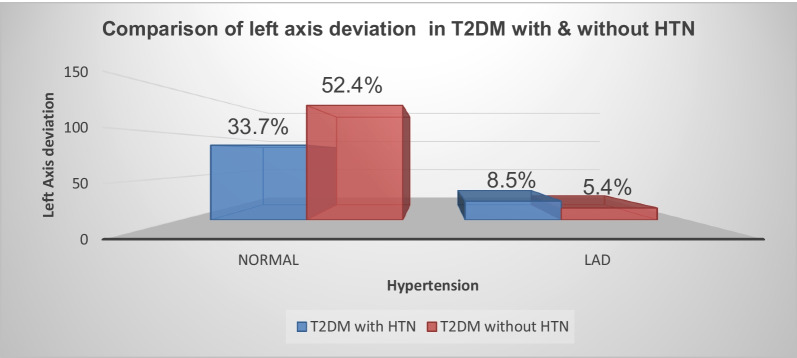
Graphical distribution of left axis deviation by hypertension among type 2 DM patients at selected referral hospitals of Amhara National regional state, 2021

The magnitude of left axis deviation in the hypertensive diabetic patient was 8.6%, while in the non-hypertensive diabetic patient was 5.4% (Fig. 1).

### Associated factors of ECG abnormality in T2DM

In the bivariable analyses at a 5% level of significance, ECG abnormality was associated with age, residence, marital status, educational status, occupation, income, body mass index, hypertension, duration of DM, fasting blood sugar, and type of DM treatment. On bivariable logistic regression, factors with *p *< 0.2 were entered into multivariable binary logistic regression. Accordingly, from socio-demographic factors, income was statistically significant with the development of ECG abnormality. The odds of developing ECG abnormality with a one-unit increase in income were reduced by 51%. Compared to those with below 2 years DM duration, those T2DM patients who had a DM duration of over 1 year were 4.5 times (AOR = 4.5, 95% CI; 1.05–18.94) more likely to develop ECG abnormality. T2DM patients who had a BMI of ≥ 25 kg/m^2^ were 2.65 times (AOR = 2.65, 95% CI; 1.17–5.98) more likely to have ECG abnormalities than those with a BMI of < 25 kg/m2. The odds of ECG abnormality in fasting blood sugar of ≥ 130mg/dl was 5.01 times (AOR = 5.01, 95% CI; 2.13–12.20) higher than those who had a fasting blood sugar of < 130 mg/dl. Those who had hypertension were 3.9 times (AOR = 1.29, 95% CI; 1.6–9.4) more likely to acquire ECG abnormality compared to their counterparts (Table 4).

**Table 4:** Sociodemographic and clinical covariates in bivariable and multivariable binary logistic regression analysis among T2DM in selected Referral Hospitals in Amhara Region, Ethiopia, 2021 (n = 258)

Variables	ECG abnormality		COR (95% CI)	AOR (95% CI)
	Yes (%)	No (%)		
Age in years	63.6±9.8	51±12.2	1.10 (1.07, 1.12)	1.02 (0.98, 1.07)
*Residence*				
Urban	74 (37.9)	121 (62.1)	1	1
Rural	42 (66.7)	21 (33.3)	3.27 (1.80, 5.95)	2.76 (0.991, 7.68)
*Current marital status*				
Married	74 (39.4)	114 (60.6)	1	1
Unmarried	42 (60)	28 (40)	2.31 (1.32, 4.05)	0.55 (0.20, 1.53)
*Educational status*				
No formal education	58 (61.7)	36 (38.3)	3.19 (1.86, 5.46)	0.96 (0.24, 3.87)
1^0^ and 2nd education	8 (53.3)	7 (46.7)	2.26 (0.78, 6.60)	3.50 (0.67, 18.20)
College and above	50 (33.6)	99 (66.4)	1	1
*Monthly income in USD*				
< 50$	69 (71.1)	28 (28.9)	1	1
50–90$	17 (53.1)	15 (46.9)	0.46 (0.2, 1.04)	0.25 (0.08–0.43)
> 90$	30 (23.3)	99 (76.7)	0.12 (0.07, 0.22)	0.51 [0.31, 0.83])*
Monthly income in USD	$82±56	$137±6	0.35 (0.26, 0 .47)	0.41(0.31, 0 .83)*
*Occupation*				
Employed	43 (35.2)	79 (64.8)	1	1
Unemployed	73 (53.7)	63 (46.3)	2.13 (1.29, 3.52)	0.58 (0.17, 2.00)
*Regular exercise*				
Yes	7 (16.7)	35 (83.3)	1	1
No	109 (50.5)	107 (49.5)	5.09 (2.17,11.97)	0.97 (0.29, 3.30)
*Duration of DM in (years)*				
< 2	11 (15.1)	62 (84.9)	1	1
2–5	20 (31.3)	44 (68.7)	2.56 (1.12, 5.88)	3.05 (0.93, 9.96)
6–10	39 (60.9)	25 (39.1)	8.79 (3.89, 19.85)	2.42 (0.93, 9.96)
> 10	46 (80.7)	11(19.3)	23.6 (9.4, 59.06)	4.5 (1.05, 18.94)*
*Body mass index kg/m*^*2*^				
< 25	32 (24.1)	101 (75.9)	1	1
25 and above	84 (67.2)	41 (32.8)	6.5 (3.75, 11.16)	2.65 (1.17, 5.98)**
*Type of DM drug*				
No medication	7 (58.3)	5 (41.7)	1	1
Oral only	99 (47.8)	108 (52.2)	0.15 (0.02, 1.23)	0.07 (0.002, 1.88)
Insulin only	6 (21.4)	22 (78.6)	0.05 (0.005, 0.45)	0.04 (0.001, 1.31)
Oral and insulin	6 (54.5)	5 (45.5)	0.17 (0.009, 2.98)	0.04 (0.0006, 2.56)
*FBS*				
< 130 mg/dl	14 (14.3)	84 (85.7)	1	1
≥130 mg/dl	102 (63.8)	58 (36.2)	10.6 (5.50, 20.24)	5.01 (2.13, 12.20)***
*HTN*				
Yes	84 (77.1)	25 (22.9)	12.3 (6.79 22.24)	3.9 (1.6, 9.40)**
No	32 (21.5)	117 (79.5)	1	1

*HTN* hypertension, *FBS* fasting blood sugar, *ISH* isolated systolic hypertension, 1 = constant

Hosmer Lemeshow Goodness of fit *p* = 0.31

*p < 0.05, **p < 0.01, ***p = 0.001

## Discussion

This study determined the patterns, and the overall prevalence of ECG abnormality, and identified its associated factors. The most common patterns of ECG abnormalities were T wave abnormalities followed by left axis deviation, sinus tachycardia, and left ventricular hypertrophy. The prevalence of ECG abnormality among this study participants was 45% which is consistent with studies in Spain (43%) [31], and Nigeria (40%) [32]. This prevalence of ECG abnormalities may be a result of the physiological condition of oxidative stress due to low glycemic control, obesity, dyslipidemia, insulin resistance, hypertension, and physical inactivity. Then, oxidative stress leads to protein kinase C signaling impairment and increased advanced glycation end products (AGE) that cause vasoconstriction, arterial inflammation, thrombosis, and atherogenesis, resulting in ECG abnormality [9–11].

However, the prevalence of this study was higher than the study done in Canada (22%) [16], Senegal (20%) [18], Netherlands (29.1%) [31], and North India 26% [33]. The reason for this difference might be due to lower ECG screening practice for T2DM patients having cardiovascular risks, like duration of DM above 15 years, above 40 years old, smoking, hypertension, and obesity [22]. Another reason might be due to failure of early detection of DM, poor medication adherence, suboptimal glycemic control, inappropriate dietary habits, and low level of regular physical activity [34]. On the contrary, the prevalence of ECG abnormality in this study was lower than in the previous studies in the USA at 60% [15], and in India at 52% [17]. This difference might be due to socioeconomic variations [35]. Moreover, this study was lower than the recently published study in Jimma medical center (Ethiopia (61%) [21]. For that discrepancy, cultural and lifestyle differences could be accountable in that person living in Jimma, Ethiopia could use substances (Khat endemic area) which would affect the health conditions.

The most commonly encountered ECG abnormality in this study was T-wave abnormality (21.1%), which is supported by previous studies in the USA [15], Nigeria [20, 36], and India [17]. The possible cause of the T-wave abnormality might be due to cardiac autonomic neuropathy (CAN) which damages autonomic nerves that innervate blood vessels and the heart resulting in resting tachycardia, silent myocardial infarction, and exercise intolerance [37, 38]. The second prevalent ECG abnormality was left axis deviation (14%). Similar reports were observed in the USA [15], Senegal [18], and India [17]. This may result from the left ventricular enlargement, left bundle branch block, myocardial infarction, and obesity-induced ventricular position change [37, 38]. The third most ECG abnormality was sinus tachycardia (9.3%), which was consistent with a study done in the USA [15], Nigeria [20, 36], and India [17]. This result might be due to cardiac autonomic neuropathy which affects the sympathetic and parasympathetic nerves that control the heart rate [37, 38].

We finally identified the associated factors of overall ECG abnormality in T2DM patients. Income, duration of DM, hypertension, fasting blood sugar, and BMI were factors significantly associated with ECG abnormality. A one $ increase in income reduces ECG abnormality by 51%. Though no previous study was conducted to show this association, the reason for this relationship might be due to increasing self-glucose monitoring practice by easily accessing the glucose monitoring materials [35], whereas those with low income may not have access to do so. The other reason might be due to increasing health care service receiving practice and the ability to have diet modifications [35].

Compared to T2DM patients who had DM duration below 2 years, those who had DM duration of over 10 years had 4.5 times more likely to develop ECG abnormality. This was supported by previous studies in the USA [15], Netherlands [19], India [33], Sub-Saharan Africa [39], Nigeria [20], and Ethiopia [21]. The reason for this result might be due to an overtime increase in blood sugar that can damage the blood vessels and the nerves that control the heart. The other reason might be due to DM-induced high blood pressure increases the force of blood through arteries and can damage artery walls. Moreover, too much cholesterol and triglyceride can form plaque resulting in the hardening of arterial walls [40] which can finally lead to ECG changes.

The odd of ECG abnormality among T2DM with hypertensive patients was 3.9 times higher than T2DM patients with no hypertension. This was consistent with the studies done in the USA [41], Netherlands [19], Spain [31], Sub-Saharan Africa [39], and Senegal [18]. This might be due to left ventricular hypertrophy as a result of higher resistance to pumping of the heart since high blood pressure enhances the peripheral vascular resistance and left ventricular afterload, and prolonged exposure to high load leads to volume and pressure-mediated structural remodeling of the left ventricle [42]. On the other hand, hypertension leads to reduce blood supply to the cardiac tissue resulting in silent myocardial ischemia [42, 43].

Compared to those with < 130 mg/dl of fasting blood sugar, T2DM patients who had ≥ 130 mg/dl were 5 times more likely to develop ECG abnormalities. This study was supported by other studies in India [33], and Netherland [19]. This may be due to the fact that transport of glucose across the cell membrane is unregulated by insulin and high glucose concentration damages cells with high intracellular glucose and glucose metabolites. These metabolites activate accessory metabolic pathways, like, as the sorbitol and protein kinase C pathways that result in the formation of oxidative free radicals and the deposition of advanced glycation end products [44–46]. However, a study showed in Jimma (Ethiopia), that fasting blood sugar was not significantly associated with ECG abnormality [21]. This difference might be due to adherence to diabetic medication, and self-glucose monitoring practice awareness creation for the patients [47].

Body mass index was another associated factor of ECG abnormality in T2DM. The odds of developing ECG abnormality in T2DM with a BMI of 25 kg/m2 and above were 2.65 times higher than those with a healthy BMI. This study was consistent with the study conducted in the Netherlands [19] and Sub-Saharan Africa [39]. The reason for this may be due to the fact that being overweight causes several hemodynamic changes like increased stroke and blood volume and an increase in left atrial and pulmonary pressure. These cause structurally altered cardiac tissue such as left atrial enlargement and remodeling, and ventricular hypertrophy. Then, ultimately, these results in obesity-induced ECG changes [48, 49]. Moreover, a high BMI may lead to derangement of lipid profiles i.e. triglyceride, cholesterol, and fatty acids that result in the formation of atherosclerotic plaque in the blood vessels. Then, ultimately it may cause vasoconstriction, thrombosis, and myocardial tissue ischemia [49–51]. Nonetheless, BMI was not significantly associated with ECG abnormalities in a study conducted in Nigeria [32]. This difference might be due to the level of physical activity, dietary modification, and lipid-lowering drug utilization [49–51].

## Strength and limitations of the study

The strength of this study was assessing the patterns of ECG abnormality and associated factors in multi-center settings, which makes it relatively more representative. But, the findings of this study should be interpreted by considering the following limitations. As the study was a cross-sectional survey, it is difficult to establish a cause-effect relationship. Moreover, the study didn’t assess the effects of lipid profiles on ECG abnormalities.

## Conclusion

Nearly, half of the study participants had electrocardiographic abnormalities. The most common ECG abnormalities were T-wave abnormalities, left axis deviation, sinus tachycardia, left anterior fascicular block, and ST-segment depression. Income, duration of diabetes, body mass index, fasting blood sugar, and hypertension were factors that have a statistically significant association with ECG abnormalities. The health sector should institute a routine ECG screening for all T2DM patients to reduce ECG abnormalities and further complications.

## Data Availability

We included all the relevant information in the manuscript but the refined dataset can be obtained from the corresponding author upon request.

## Abbreviations

BMI: Body Mass Index
CAD: Coronary artery disease
DM: Diabetes mellitus
ECG: Electrocardiography
FBS: Fasting blood sugar
LVH: Left ventricular hypertrophy

## Declarations

### Patient and public involvement

Patients were not formally involved in developing this specific study design.
